# Diabetes self-management education after pre-selection of patients: design of a randomised controlled trial

**DOI:** 10.1186/s13098-016-0199-4

**Published:** 2016-12-20

**Authors:** Rimke C. Vos, Nathalie W. D. Eikelenboom, Maarten Klomp, Rebecca K. Stellato, Guy E. H. M. Rutten

**Affiliations:** 1 Department of General Practice, Julius Center for Health Sciences and Primary Care, University Medical Center Utrecht, Huispostnr. STR.6.131, P.O. Box 85500, 3508 GA Utrecht, The Netherlands; 2 DOH Care Group, Tilburgseweg-West 100, P.O.Box 7140, 5652 NP Eindhoven, The Netherlands; 3 Department of Biostatistics and Research Support, Julius Center for Health Sciences and Primary Care, University Medical Center Utrecht, Huispostnr. STR.6.131, P.O. Box 85500, 3508 GA Utrecht, The Netherlands

**Keywords:** Type 2 diabetes mellitus, Self-care, Psychological adaptation, Cost-benefit analysis, Quality of life, Nurses practice patterns, Education

## Abstract

**Background:**

Many self-management programs have been developed so far. Their effectiveness varies. The program ‘Beyond Good Intentions’ (BGI) is based on proactive coping and has proven to be (cost-) effective in achieving reductions in BMI and blood pressure in screen-detected type 2 diabetes patients up until nine months follow-up. However, its long-term effectiveness in people already known with diabetes is lacking. In addition, its (cost-) effectiveness might increase if people who are likely not to be benefit from the program are excluded in a valid way. Therefore it was aimed to investigate the long-term effects of the educational program BGI on cardiovascular risk, quality of life and diabetes self-management behaviour in a pre-selected group of patients known with type 2 diabetes up to 5 years.

**Methods:**

Randomised controlled trial with 2.5 year follow-up. Adults (≤75 years) with a type 2 diabetes duration between 3 months and 5 years will be included. With the use of a self-management screening tool (SeMaS) their potential barriers of self-management due to depression and/or anxiety will be determined. Based on the results of the SeMaS selection patients will be randomised (1:1) to the BGI-group (n = 53) or the control-group (n = 53). In addition to receiving usual care, patients in the BGI-group will follow the 12-week theory-based self-management program and a booster session a few months thereafter. The control-group will receive care as usual. The primary outcome is change in Body Mass Index after 2.5 years follow-up. Secondary outcomes are HbA1c, lipid profile and systolic blood pressure, (diabetes) quality of life, level of physical activity, dietary intake and medication adherence and proactive coping. Cost-effectiveness will be based on total use of health care resources during the entire study period. Difference between groups in change over time will be analysed according to intention-to-treat analysis.

**Conclusions:**

By differentiating between patients who will and those who are likely not to benefit from the educational program, a more (cost-) effective self-management program might be designed, also on the long-run.

*Trial registration* NTR 5330

## Background

Diabetes self-management forms a major part of diabetes treatment and involves several complex behaviours, such as adhering to a treatment regimen, monitoring symptoms, maintaining a healthy lifestyle and managing the impact of the disease on one’s life. People with type 2 diabetes mellitus (T2DM) are expected to take more and more responsibility in this, but for many of them self-management is difficult to incorporate in daily life and not all patients will possess the necessary skills for this. The motivation to initiate changes in behaviour is often present, but patients do not know what needs to change or maintain the achieved behavioural lifestyle when barriers are encountered. We developed the Beyond Good Intentions (BGI) program to enhance the ability of patients to act upon their intentions. It is based on the theories of self-regulation and proactive coping [1], assuming that people should control and direct their actions in order to meet their goals. Pro-active coping refers to efforts undertaken in advance of a potentially stressful event to prevent or modify its form before it occurs [1]. BGI has proven to be effective in patients with screen-detected T2DM up until 9 months follow-up [2]. Based on a computer-based simulation model to project long-term health benefits and cost-effectiveness, BGI was among the four diabetes self-management programs worldwide, with a minimum follow-up of 12 months, with the best cost-effectiveness ratio [3]. However, real, not modelled, long-term data on (cost-) effectiveness of BGI are lacking and also its effectiveness in people who are known with T2DM and not screen-detected, is unclear.

Group based educational programs for diabetes self-management can improve blood glucose, blood pressure, body mass index (BMI) and the requirement of blood glucose lowering drugs; however, the long-term results were varying [4]. In Italy, non-insulin-treated T2DM patients known with diabetes for at least 1 year were randomised to either group or individual care in a hospital-based setting. After 4 years clinical, cognitive, and psychological outcomes were better in the group based treatment [5]. In the DESMOND trial, greater improvements in weight loss and smoking cessation were found in the intervention group at 1-year-follow-up, but at 3-year-follow-up this effect was no longer apparent [6, 7]. In general, completers of a diabetes self-management intervention study are significantly more highly educated than dropouts [8]. This indicates that interventions to improve self-management should be tailored to different categories of patients. The ‘one size fits all’ principle in diabetes education research is outdated. To estimate a priori the chance of successful self-management of a patient, the self-management screening tool (SeMaS) can be used [9]. With SeMaS possible barriers for self-management due to depression and/or anxiety can be detected. Both depression and anxiety can interfere with the effectiveness of a self-management program at that moment for a particular person and should be dealt with before following the program. Besides, individuals with already high self-management capabilities are likely to have no additional benefit from an educational course [10–12].

We hypothesise that by differentiating between patients who may and those who may not benefit from a self-management program, a more (cost-) effective self-management education program can be designed which is also effective on the long-run. Therefore, the aim of the current study is to investigate the cost-effectiveness and long-term effects of the educational program BGI on cardiovascular risk, quality of life and diabetes self-management behaviour in a preselected group of known T2DM patients.

## Methods

### Setting

In Dutch primary care, most patients (80–85%) are treated within disease management programs, contracted by so called care groups. These care groups are responsible for the organisation, coordination and delivery of diabetes care in primary care [13, 14]. Three care groups with 43 general practices (both group and single handed practices, with 89 general practitioners) in the southern part of the Netherlands participate in the current study.

### Study design and selection criteria

The study is designed as a parallel randomised controlled trial with 2.5 years follow-up and will follow the CONSORT criteria for experimental designs [15]. Along with the BGI self-management education program (obviously only for the intervention group) all participants will receive diabetes care according to the guidelines of the Dutch College of General practitioners [16].

#### Inclusion criteria

- Adults, aged ≤75 years.
- Diagnosed with T2DM between 3 months–5 years ago.

#### Exclusion criteria

- High self-management capability on all SeMaS domains (see below).
- High scores on the SeMaS domains ‘Anxiety’ or ‘Depression’ (see below).
- Insufficient cognitive functioning.
- Insufficient understanding of the Dutch language to follow instructions and to complete questionnaires.

### Ethical approval

The study was approved by the Medical Ethical committee of the University Medical Centre Utrecht and registered at the Dutch Trial Register (Netherlands Trial Register NTR5330).

### Recruitment

The electronic medical records from the people with T2DM from the participating general practitioners are scrutinised to find eligible T2DM patients. All patients who meet the inclusion criteria will be informed about the possibility to follow a group-based diabetes self-management education program with pre-selection (see further), emphasising that they will be only offered an educational program they are likely to benefit from. People can indicate on a response card whether or not they want to receive oral information about the study. If yes, a research team member will give the patient a telephone call to discuss the study. If the patient is informed and understands the information, informed consent will be completed. Once the patient has given informed consent the pre-selection will take place.

### Pre-selection

The pre-selection procedure to assess a patient’s capabilities of self-management will be performed with the use of the SeMaS, a self-management screening tool which is validated in T2DM patients and sent to the patients home [9]. The SeMaS is a 27 item questionnaire covering self-management abilities of five psychosocial domains [locus-of-control (3 items), self-efficacy (2 items), social support (1 items), coping (9 items), anxiety (4 items) and depression (3 items)], three skills items (computer, group and self-care) and two other items including perceived burden of disease [9]. All separate items range from 0 to 3 on a 4-point Likert scale (locus-of-control, self-efficacy, coping, skills items) or from 0 to 2 on a 3-point Likert scale (social support, anxiety, depression). For all items higher scores represent higher capability, except for those in the anxiety and depression domain where higher score represent more barriers. The perceived burden of disease is scored on a visual analogue scale from 0 to 10. Patients with high scores on the domains anxiety (score >4 from total 8) and/or depression (score >3 of total 6) will be excluded during the pre-selection. Those patients first need to deal with their mood disturbance before they are able to improve their self-management skills. Patients without barriers for self-management, so with maximal self-management capacity (highest possible scores for the locus-of-control, self-efficacy, social support, coping and for anxiety ≤4 and for depression ≤3) were also excluded, since they would not benefit from the educational course. The general practitioner (GP) will be informed about the SeMaS scores of all participants.

### Randomisation

Patients who are eligible after the pre-selection will be randomised (1:1) based on computer-generated random numbers, with sealed, opaque, sequentially numbered allocation envelopes. All patients entering the trial receive a random number from the coordinating research centre (Julius Center). Each number corresponds to either ‘BGI course’ (BGI-group) or ‘no BGI course’ (control-group). As patients will attend a course in the intervention group, it is not possible to blind participants for the treatment allocation. Patients from the same practices and general practitioners will attend a different course, so no large cluster-effect is expected.

### Intervention: beyond good intentions (BGI)

This study elaborates on the existing self-management program BGI [2, 17, 18]. It lasts 12 weeks and consists of one initiating 30-min individual session and four 2.5-h group sessions. In addition, one booster-session is given a few months after the last group session. The course structure is as follows:

#### Individual session

The patient’s knowledge, attitudes and ambivalence with regard to diabetes management will be discussed. For this purpose the ‘Diabetes Profile’ was specifically designed to help patients gaining insight in the risk factors associated with their diabetes. With a colour scheme, from green to red, their risk for long-term complications is presented. Patients are encouraged to set goals to work on. The trained practice nurse (see further) discusses glycaemic control, BMI, blood pressure and lipids profile with the patient. Patients are asked to formulate a first goal before the first group session and will receive the educational material that will be discussed during the group sessions.

#### Group sessions

The group sessions are held in groups of six to ten participants and take place every two to three weeks in a location nearby the patients’ homes. The first three sessions will cover topics that are relevant for all patients: physical activity, healthy diet and medication adherence (what do guidelines tell, why it is important to follow these guidelines and how to do it, what are known barriers in this respect, what might facilitate compliance, followed by individual goals to improve). In the fourth session patients will be able to work on a personally relevant goal.

Every group session starts with the introduction of the topic, patients are asked to share their opinions, emotions and experiences regarding the topic. Subsequently, patients write their own individual action plan, according to the proactive coping five-step plan:

- Step 1: Formulate a concrete and attainable goal.
- Step 2: What are necessary conditions and possible barriers?
- Step 3: What may be alternative strategies for crossing these possible barriers?
- Step 4: Formulate the final plan: what, how, where, and when?
- Step 5: Indicate how the progress will be evaluated and when you are satisfied, the plan is discussed with the group to improve the final plan.

During the weeks in between the groups sessions patients will practice with their individual action plane, which will be evaluated during the next meeting.

In case of diabetes-related questions, patients are encouraged to collect additional information by themselves, e.g. on the Internet or by referring them to another professional like a physiotherapist or dietician. This way, patients are encouraged to take responsibility for their disease.

#### Booster session (evaluation)

In this session the past months will be discussed and goals will be evaluated. New goals will be set. In addition, the trainers will motivate the participants to bring up a topic for the booster.

### Nurse training

The BGI course will be given by trained practice nurses (n = 3). They receive two 3-h training sessions from the psychologist who is familiar with the BGI training (Train-the-trainer principle). During the training they will work with the five-step proactive coping plan and will be taught on the principles of the self-regulation and pro-active coping theory. The practice nurse should have the role of facilitator and coach, stimulating participants to support each other and become self-confident in seeking information.

### Control group

As stated above, participants in the control group will receive similar diabetes care as those in the intervention group, according to the guidelines of the Dutch College of General Practitioners [16]. The only difference between both groups will be the additional BGI course.

### Outcome measures

#### Primary outcome measure

The primary outcome measure will be the same as in the original BGI study for comparison reasons and is therefore; change in BMI (difference of 0.77 kg/m^2^ is considered clinical relevant) between baseline and follow-up (2.5 years after the start of the BGI program).

#### Secondary outcome measures

The secondary outcomes will be change between baseline and 2.5 years follow-up of:

1. Clinical measures: systolic blood pressure, HbA1c, lipid profile (total cholesterol, LDL, HDL, triglycerides);
2. Diabetes self-management behaviour (self-care, physical activity, dietary intake, medication adherence);
3. Proactive coping;
4. Quality of Life;
5. Cost-effectiveness of the intervention (total health care resources use during the entire study period).

### Measurements

#### Clinical measures

In both groups, BMI, systolic blood pressure, HbA1c and lipid profiles will collected from the electronic medical record at baseline and 2.5 years follow-up. Clinical measures in-between will also be extracted from the electronic medical record of the GP.

#### Questionnaires

Participants will be asked to complete questionnaires to measure diabetes self-management behaviour, proactive coping and quality of life at baseline and 2.5 years follow-up, with the following measures.

#### Diabetes self-management behaviour

- The SDSCA, revised according to Toobert et al. [19], includes eleven items assessing several aspects of the diabetes regimen: general diet, specific diet, exercise, blood glucose testing, foot care, and smoking. Ten items are rated on an 8-point Likert scale, measuring how many days an activity is performed in the last week. One item measures smoking status (yes/no) and the amount of cigarettes smoked in the last week. Each of the domains is measured separately.
- The PASE questionnaire [20] includes 15 items, which cover leisure time physical activities (six items), household physical activities (six items), and work related physical activities (three items). Items differ in scoring range and are translated to the time spent on each activity into a composite score which reflects the amount of energy expended.
- The ‘Kristal food habits questionnaire’ [21] consists of 22 items assessing patient’s habits with regard to fatty food intake, dietary products and fruit and vegetable intake. The items differ in scoring ranges.
- The MARS-5 [22], is a brief 5-item instrument which assesses the degree to which patients forget medication, stop taking their medication or change dosages. Items range from 1 (always–never adherent) to 5 (never–always adherent).

#### Proactive coping

Proactive coping will be measured with the ‘Proactive Diabetes Management Inventory’ (PDMI) [17], designed to evaluate the success of our course in getting patients to implement the five-step plan in their self-care. The instrument includes 7 statements covering different aspects of the five-step plan. The first six items assess whether patients think about their goals beforehand in a proactive fashion; that is, do they anticipate the conditions needed to reach their goal and do they anticipate barriers they may encounter along the way? Items 7–12 assess whether patients anticipate and plan the strategies needed to achieve their goal. Finally, Items 13–17 assess whether patients monitor their progress and strategies and adapt these accordingly. The items range from 1 (definitely not) to 5 (definitely) [17].

#### Quality of life

The EuroQol EQ-5D, Short-Form 36 (SF36) and a diabetes-specific quality of life questionnaire, namely the Audit of Diabetes-Dependent Quality of Life (ADDQol) will be used to measure health-related quality of life as well as health status.

- The EQ 5D is a generic quality of life questionnaire, consisting of a Visual Analogue Scale (EQ 5D-VAS) and a classification system (EQ 5D-Profile) [23]. The EQ 5D-Profile covers five domains: mobility, self-care, usual activities, pain/discomfort and anxiety/depression of health, each with three levels of functioning: level 1, no problems; level 2, some problems; level 3, severe problems. The EQ-5D health utility scores range from −0.33 to +1.00 and were computed using the Dutch tariff as described by Lamers et al. [24, 25]. A score of 0 is equal to death, whereas 1 indicates full health. Negative values represent a health state worse than death. The EQ 5D-VAS is a graduated, vertical line, anchored at 0 (worst imaginable health state) and 100 (best imaginable health state). The patient is asked to mark a point on the EQ 5D-VAS that best reflects his/her actual health state.
- The SF-36 generates a profile of scores on eight dimensions of health status: Physical Functioning (10 items), Role Physical (4 items), Bodily Pain (2 items), General Health (5 items), Vitality (4 items), Social Functioning (2 items), Role-Emotional (3 items) and Mental Health (5 items). These scales can be summarised in Physical Health and Mental Health. The 36 items differ in the scoring ranges [26].
- The ADDQoL, measures the perceived impact of diabetes on the quality of life; it includes 19 items, ranging from −3 to 3 on different questions, with 0 as the neutral score. Scores below 0 reflect a negative influence of the item on quality of life, and those above 0 reflect positive influences. The impact scores are weighted (impact rating x importance rating), so the actual scores per item can range from −9 to 9 [27].

#### Cost-effectiveness

Data on health care resources use during the whole study period will be extracted from electronic medical records from the general practitioners and for health status the EQ 5D questionnaire will be used at baseline and after 2.5 years follow-up.

### Sample size

In our previous study, the self-management intervention BGI achieved a significant BMI reduction of 0.77 after 9 months in screen-detected T2DM patients (BMI −0.39 in the intervention group vs. +0.38 in the control group) [2]. For the current study a minimum sample size of 88 (44 per group) is required to achieve 80% power to detect a difference of 0.77 in a design with 6 repeated measurements of BMI (weight is measured every three months as part of usual diabetes care) having a AR (1) covariance structure when the standard deviation is 1.7, the correlation between subsequent observations on the same subject is 0.70, and alpha 0.05. With an expected drop-out rate of 20%, we need to include a total of 106 patients.

### Statistical analyses

The efficacy analysis will be performed by intention-to-treat analysis, including all patients from whom a follow-up assessment is available. If necessary a multiple imputation technique will be used for missing data. The primary outcome will be analysed by using Generalized Estimated Equations (GEE) for repeated measures, with baseline BMI as covariate and time, intervention and the interaction of time and intervention as fixed variables. The secondary outcomes will be analysed with ANCOVA on the change from baseline to follow-up. In addition differences in characteristics (e.g. age, sex, education level) of participants and non-participants will be evaluated.

### Cost-effectiveness

Lifetime costs and health effects (based on the EQ 5D) will be estimated using a modified probabilistic diabetes model. This validated model has been used before and is described in more detail elsewhere [28–30]. In brief, the model simulates the natural history of type 2 diabetes and calculates costs and quality-adjusted life years (QALYs) for Dutch T2DM patients [30]. The model includes a health state for cardiovascular disease (CVD; myocardial infarction, stroke, peripheral arterial disease), microvascular complications (end-stage renal disease, retinopathy and neuropathy), uncomplicated type 2 diabetes and death. The model computes the occurrence of the above-mentioned diabetes-related complications and the excess mortality due to diabetes. Based on the estimated events and prevalence of complications, it computes diabetes-related lifetime medical costs and QALYs.

To calculate lifetime costs and outcomes, each health state is assigned a value in terms of medical costs and utility (health-related quality of life), and this value is multiplied by the prevalence of the health states over time.

In addition to the model input data described above, medication costs of glucose-lowering drugs, anti-hypertensive medication and cholesterol-lowering drugs used during the total follow-up period will be included in the cost calculation. Costs of the BGI-course and total health care resources (including consultations of the dietician) use during the whole study period will also be included in the cost calculation. These costs will be extrapolated to estimate lifetime medication costs, assuming that the cost difference between the BGI-group and the control-group remains constant over time.

The model will calculate two lifetime health outcomes (life years, QALYs) and costs for each patients (discounted and undiscounted). To correct for confounding and improve model estimates of the difference in outcome between the BGI-group and the control-group, the following baseline covariates will be used: age, sex, duration of diabetes, history of cardiovascular disease, smoking, HbA1c, systolic blood pressure, total cholesterol and HDL cholesterol.

The primary outcome of the cost-effectiveness analyses will be the cost-effectiveness of BGI versus control, expressed as the incremental cost-effectiveness ratio (ICER), calculated by dividing the incremental costs by the incremental QALYs or incremental life years. Uncertainty surrounding the cost-effectiveness ratios as calculated from the model will be expressed using a cost-effectiveness plane. A cost-effectiveness acceptability curve will be created to determine whether implementation of BGI is cost-effective given different thresholds of willingness to pay for a QALY (e.g. a threshold of € 20.000 per QALY).

## Discussion

In diabetes self-management education the ‘one-size-fits-all’ approach is not very effective. Not all patients will possess the necessary self-management skills. While a certain motivation and perseverance may be needed to change one’s behaviours, successful maintenance ultimately depends on one’s ability to anticipate and deal with a wide range of potential stressors that challenge diabetes management and long-term health. This is the central tenet of proactive coping, a self-regulatory model which describes the steps people take in pursuit of their long-term goals. From our previous research we know that proactive coping may be a better predictor of good long-term self-management than either intentions or self-efficacy [18]. The current study will add knowledge about the program in three different aspects: its 2.5 year (cost-) effectiveness and the impact of pre-selection of patients. This knowledge will be of importance, because to deal with the global diabetes epidemic, cost-effective self-management education programs that are also effective on the long run are needed.

## Abbreviations

ADDQoL: Audit of Diabetes-Dependent Quality of Life
BGI: beyond good intentions
BMI: body mass index
CVD: cardio vascular disease
EQ-5D: Euro QoL 5D
EQ-VAS: Euro QoL visual analog scale
DOH: De Ondernemende Huisarts
QALY: quality-adjusted life years
PASE: physical activity scale for elderly
PDMI: proactive diabetes management inventory
PoZoB: Praktijkondersteuning Zuidoost Braband
SDSCA: summary of diabetes self-care activities measure
SeMaS: self-management screening tool
SF-36: short-form 36
SGE: Stichting Gezondheidscentra Eindhoven
T2DM: type 2 diabetes mellitus
